# An Efficient Model for a Vast Number of Bird Species Identification Based on Acoustic Features

**DOI:** 10.3390/ani12182434

**Published:** 2022-09-15

**Authors:** Hanlin Wang, Yingfan Xu, Yan Yu, Yucheng Lin, Jianghong Ran

**Affiliations:** 1 Key Laboratory of Bio-Resources and Eco-Environment (Ministry of Education), Sichuan University, Chengdu 610065, China; 2 School of Informatics, University of Edinburgh, Edinburgh EH8 9YL, UK

**Keywords:** bird calls, deep learning, species identification, avian biodiversity

## Abstract

**Simple Summary:**

Identifying bird species is very important in bird biodiversity surveys. Bird vocalizations can be utilized to identify bird species. In this paper, we utilized massive amounts of data of bird calls and proposed a novel, efficient model for identifying bird species based on acoustic features. A novel method was proposed for audio preprocessing and attention mechanism embedding. Our proposed model achieved improved performance in identifying a larger number of bird species. Our work might be useful for bird species identification and avian biodiversity monitoring.

**Abstract:**

Birds have been widely considered crucial indicators of biodiversity. It is essential to identify bird species precisely for biodiversity surveys. With the rapid development of artificial intelligence, bird species identification has been facilitated by deep learning using audio samples. Prior studies mainly focused on identifying several bird species using deep learning or machine learning based on acoustic features. In this paper, we proposed a novel deep learning method to better identify a large number of bird species based on their call. The proposed method was made of LSTM (Long Short−Term Memory) with coordinate attention. More than 70,000 bird−call audio clips, including 264 bird species, were collected from Xeno−Canto. An evaluation experiment showed that our proposed network achieved 77.43% mean average precision (mAP), which indicates that our proposed network is valuable for automatically identifying a massive number of bird species based on acoustic features and avian biodiversity monitoring.

## 1. Introduction

As indicators of biodiversity, birds are always worthy of being surveyed for biodiversity conservation [1,2]. It was verified that bird calls are relatively stable as they present observable acoustic features among bird species [3]. Many kinds of research considered identifying bird species or individuals by analyzing their calls manually [4], such as analyzing the waveform, spectrogram, Mel−spectrogram [5,6], and Mel−frequency cepstral coefficients [7] using bird−call clips. Nonetheless, it was time−consuming if the audio data expanded a lot, which is inefficient and biased in subjective ways. Several automated methods were presented to reduce labor costs, yet still had room to be improved.

Spectrograms are representations of audio signals that show the prominence of sinusoidal components in sliding time frames [8,9]. They are basically generated by discrete Fourier transform [10,11] since any audio signal can be superimposed using multiple sinusoids. DFT converts finite sequence samples of audio signals into a same−length sequence of equally−spaced discrete−time Fourier transform (DTFT), which is a complex-valued function of frequency [12]. Thus, spectrograms render frequencies and intensity (or amplitude) distribution along with sliding time frames. Essentially, spectrograms are full of fine−grained features of frequencies that can be extracted and utilized for subsequent acoustic analysis [13]. However, perceived intensity is not proportional to signal magnitude [14]. Amplitude in an audio signal, which also equals volume, is just a representation of physical magnitude, and loudness is the semantic metric from which human beings can perceive the intensity of sound [15]. In this case, the spectrogram is not a kind of semantic spectrum from which the semantic features cannot be potentially extracted. Mel−spectrogram [5], full of semantic features since it is fitted well with human hearing, is formed by assigning proper filter banks to the spectrogram. From the features of the Mel−spectrogram, it is easier to distinguish the differences in calls of bird species than using features from the spectrogram. MFCCs are a further transformation of a spectrum that is transformed by discrete cosine transformation [16] from Mel−spectrogram, which is a kind of high−dimensional concentration of Mel−spectrogram, and the number of orders is optional. In MFCCs, the bands are equally spaced on the Mel scale, which is closer to the response of the human auditory system than the linearly spaced bands used in the normal spectrum. This frequency warping can allow for better representation of sound [17]. Another transformation of an audio signal is the Chirplet−spectrogram, which can be regarded as the combination of wavelet−spectrogram and Mel−spectrogram [18]. These types of a spectrum of bird calls, especially MFCCs, were widely used as original input in bird species identification tasks. MFCCs have fewer feature dimensions than Mel-spectrogram, which is more conducive to training traditional machine learning algorithms.

Comparing the MFCCs and inversed MFCCs, Ramirez et al. [19] utilized the hidden Markov model (HMM) [20] to classify five bird species. The results showed that the model reached the highest mean accuracy of 58.9%. Shan et al. [21] proposed the MFCC−HMM model and achieved a recognition rate of 90.47% among the six birds. As a probabilistic graph model [22], HMM has demonstrated its impressive performance in bird call recognition because bird calls possess certain temporal features [23]. HMM leverages contextual information through hidden states, which is a huge advantage over traditional machine learning algorithms [24]. HMM is a statistical model of sequential data that has been successfully used in many applications, including speech recognition and modeling of biological sequences [25]. Apart from HMM, an MFCCs−based method was proposed to identify bird species using the K−means algorithm [26]. Chou et al. computed the mean, QI, and QE from MFCCs, combined them into a feature matrix, and utilized the fuzzy c-mean clustering method (FCM) with LDA to identify bird calls out of 420 bird species. The results showed that the highest accuracy obtained by FCM with LDA based on the combined feature was 84.34% when a specific threshold was given. Other related studies included random forest [27] and support vector machine [28]. Prior studies mainly considered implementing traditional machine learning algorithms, which need to be fine-tuned by researchers.

Deep learning [29] gives a chance to distill these acoustic features efficiently. It is part of a family of machine learning methods based on artificial neural networks (ANNs) with representation learning. Deep learning can be used to extract more information from data when compared to shallow learning techniques and has been showing impressive results in the last few years [30,31]. It requires relatively little pre−processing compared to other classification algorithms (e.g., SVM, FLDA, Random Forest, and Bayesian Inference). The networks learn to optimize the filters through automated learning, whereas in traditional algorithms, these filters are hand−engineered. This independence from prior knowledge (e.g., Naïve Bayes needs a lot of prior probabilities) and human intervention in feature extraction is a major advantage [32]. As a branch of deep learning, convolutional neural networks (CNNs) [33] have demonstrated their strong performance in image recognition and speech recognition. It has been widely used in bird song recognition. Koops et al. [34] utilized the Mel−frequency cepstral coefficients (MFCCs) of bird call clips to generate the classifier using CNN and achieved 73% accuracy. Tóth and Czeba [35] utilized the spectrogram of bird song clips to identify bird species and reached 40% mAP. Xie et al. [36] proposed KD−CLDNN to identify recordings of 20 bird species and achieved 97.5% accuracy. Piczak [37] utilized the ensemble deep convolutional neural network model to identify 999 bird species and achieved 41.2% mAP. Zhang et al. [38] proposed a method that leveraged 3−D convolution kernels to extract both the positional and temporal features from Mel−spectrogram. It reached 97% mAP in identifying four bird species. Sprengel et al. [39] processed background noise through image processing and input it into CNN. The mAP score of major species was 0.686. Kumar et al. [40] leveraged transfer learning to efficiently train the CNN model to identify bird sounds in different environments. Effendy et al. [41] proposed GamaDet and GamaNet, which leverage CNN to identify bird sounds and assess forest quality. The GamaDet is an integrated device that can record, store, and process bird sounds as well as assess and display forest quality index and identification results of bird species, while the GamaNet is an online digital product that can output graphical reports of identification results of bird species and forest quality index. By enhancing the CNN with Residual Neural Network (ResNet) [42], Kahl et al. [43] proposed BirdNET, which aims to identify 984 bird species based on their calls. BirdNET achieved 79.1% mAP among these bird species.

As another branch of deep learning, recurrent neural networks (RNNs) [44] leverage temporal context from input features. Qiao et al. [45] utilized the encoder and decoder based on RNN to learn the higher representations of features of bird sound. This study showed that the model achieved better unweighted recall performance by regarding the higher representations of features as input for the subsequent machine learning algorithms.

Cross−modalities method is another research direction in identifying bird calls. Zhang et al. [46] combined the STFT−spectrogram, Mel−spectrogram, and Chirplet−spectrogram as input features and used CNN to identify 18 bird species. The results demonstrated that the best mAP reached 91.4%. Conde et al. [47] designed a process that introduced the ensemble model by integrating multiple CNN models and the SVM algorithm. The results showed that the best F1−score reached 69.9% on BirdCLEF 2021 dataset [48]. Cakir et al. [49] proposed a method combining CNN with RNN to realize automatic detection of bird calls and obtained an 88.5% AUC score on evaluation data. Gupta et al. [50] novelly proposed a deep learning approach integrating CNN with CNN or RNN of LSTM, GRU, and LMU. The approach systematically compared the multiple hybrid models of CNN with CNN, or CNN with RNN. The results demonstrated that the CNN plus GRU achieved the best average accuracy of 67% over 100 bird species.

The studies above show that the bird species identification method based on deep learning is effective. The number of target bird species is related to the performance of the model. The more bird species the model identifies, the more possibility of better defective performance. Most relevant studies mentioned above can be classified into two genres: one for a large amount of bird species identification with relatively insufficient performance; the other for a small number of bird species identification with relatively high performance. In addition, cross−modalities integration is a good way to leverage acoustic features. In this paper, we proposed an efficient deep learning method for identifying massive bird species based on their acoustic features. Our main contributions can be summarized as follows:

1. We proposed a novel method for preprocessing audio samples before sending them to the training stage.
2. We fused Mel−spectrogram and MFCCs as input features and discussed the impact of orders of MFCCs on the performance of the model.
3. We first introduced the coordinate attention module in identifying bird species.
4. A robust deep neural network based on LSTM for bird call identification was proposed. Seven performance metrics were used to evaluate models.

## 2. Materials and Methods

### 2.1. Data

**Figure 1:**
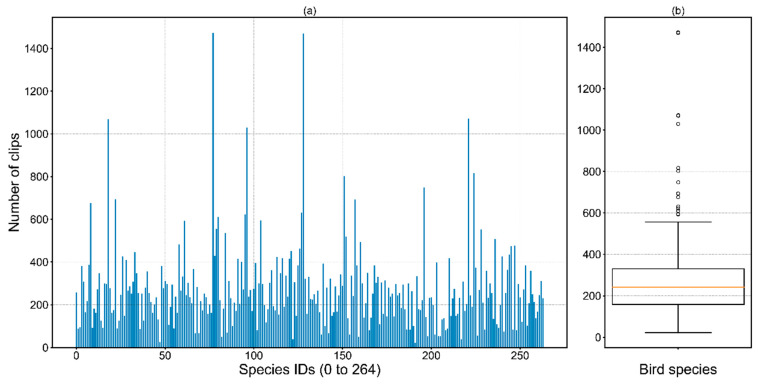
Distribution of bird call clips. (**a**) Overview of distribution of bird call clips, (**b**) Statistics: *Num_max_* = 1472, *Num_min_* = 23, *Num_std_* = 196, *Num_avg_* = 273.

A total of 72,172 bird call samples, including 264 bird species (Figure 1), were acquired from Xeno−Canto [51]. These audio samples are in 16−bit wav format with a 16 kHz sampling rate. There are 273 samples of each species on average, which might be helpful for model training.

#### Data Preprocessing

**Figure 2:**
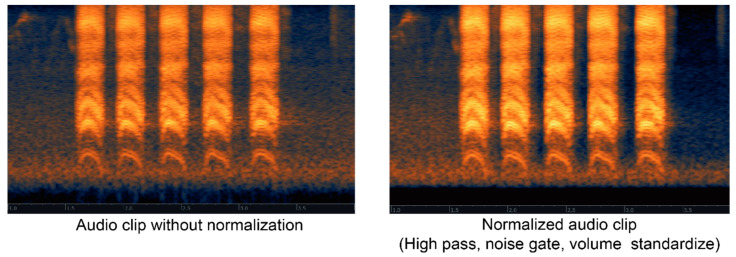
Comparison of the audio clips (*Corvus brachyrhynchos*). High pass filters the redundant low frequencies; noise gate suppresses the background noise; volume standardizer gains the maximum volume to zero decibels.

The original audio data were full of invalid signals concentrated in low frequencies; potential interference of model training. Thus, each sample was assigned a generalized high−pass filter to remove the low frequencies, especially between 0~150 Hz. As the signal−to−noise ratio (SNR) plays a crucial role in quantifying the quality of audio samples [52], a gate function with a specified threshold was implemented to eliminate the redundant background noise (Figure 2). The way birds make a call is the primary reference for identifying species. Therefore, a syllable is a key to identifying bird species. An audio clip is considered valid if it contains at least one syllable, which conventionally lasts for about 50~500 ms. Except for the rhythmic syllable interval of birds call, all audio samples were trimmed compactly to remove the sound of silence. To reduce the risk of being biased to an audio clip in a certain length and amplitude, we normalized all of the audio samples into standard amplitude clips with 10 s in which the peak signal is zero decibels.

### 2.2. Methodology

#### 2.2.1. Mel−Spectrogram and MFCCs Generating

A spectrogram is in the shape of a two−dimensional matrix, where the first dimension is pointed to the segments of frequencies, and the second dimension is pointed to the indices of timings. Since the spectrogram is rendered considering both the maximum signal and minimum signal, it is necessary to normalize the ratio of signal and noise. For instance, if the ratio of signal and noise is too low, it indicates that there is too much noise in the presence and will interfere with the valid signal, and the spectrogram will be inappropriate to express the distribution of sound intensity, as the color density of the area of signal in the spectrogram is suppressed by noise.

**Figure 3:**
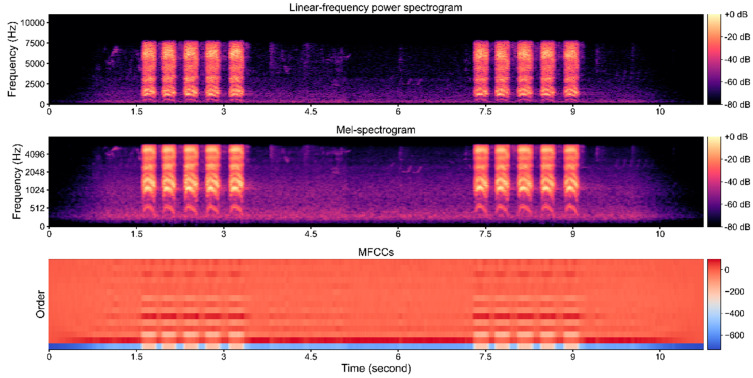
Spectrogram and MFCCs of *Corvus brachyrhynchos*.

Here we considered both Mel−spectrogram and MFCCs as input for the deep learning model (Figure 3). Mel−spectrogram was distilled from spectrogram by assigning 128 Mel filter banks [53] to spectrogram generated by discrete Fourier transform (DFT) [10]. We assigned a window size of 400 time points and a hop length of 200 time points for Mel-spectrogram generating. Each window occupies 0.025 s and the step time is 0.0125. Therefore, in a single 10−s bird call clip with a 16 KHz sampling rate, 800 frames of DFT were generated in total. DFT on specified frequency is defined as:

$$X_{k}=\overset{N-1}{\underset{n=0}{\sum }}x_{n}\cdot e^{-j2\pi \frac{k}{N}n}$$

$$k=\frac{sr}{2N}$$

where $X_{k}$ is the total magnitude of the k−th frequency bin, N is the window size, $x_{n}$ denotes the amplitude of the original analog audio signal at the time n, $e^{-j2\pi \frac{k}{N}n}$ is the analyzing function of sinusoids, k represents the indices of frequency bins and is determined by half of the window size according to the sampling theorem [54]. The Mel−spectrogram was generated by feeding the prior results of DFT into Mel scale filter banks, which can be formulated as:

$$f_{mel}=2595{log}_{10}(1+\frac{f}{700})$$

where $f_{mel}$ denotes the Mel frequency and the f is the original frequency. MFCCs were calculated on the decibel−scaled Mel−spectrogram by assigning discrete cosine transform (DCT) to Mel−spectrogram. The orders of MFCCs were set to 20, which was empirically proved to be efficient (see Section 3.3.1.) for demonstrating the distribution of features. Finally, we concatenated Mel−spectrogram and MFCCs to form a matrix of 148 × 801.

#### 2.2.2. Coordinate Attention Module Embedding

**Figure 4:**
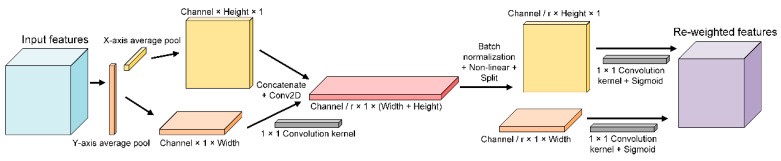
Implementation of the coordinate attention.

The coordinate attention (CA) [55] module (Figure 4) was implemented on input features seeking more channel−wise and spatial contextual information in both horizontal and vertical directions, respectively. As shown in Figure 4, the input features were pooled along the horizontal (*Y*−axis) and vertical (*X*−axis) directions to obtain two two−dimensional feature maps, respectively. These feature maps were concatenated and computed by 1 × 1 convolution for more channel−wise interactions. Subsequently, the concatenated feature map was normalized, followed by activation, and then spatial−wise split into **f**^h^, **f**^w^, respectively. Another corresponding pair of 1 × 1 convolution kernel whose number of channels is equal to the original input features was implemented on **f**^h^, **f**^w^ yielding the final pair of attention weights for horizontal and vertical directions, respectively. Note that sigmoid functions were activated on 1 × 1 convolution for non−linear representation. The re-weight process can be formulated as:

$$y_{c}\left(i,j\right)=x_{c}\left(i,j\right)\times g_{c}^{h}\left(i\right)\times g_{c}^{w}\left(j\right)$$

where i denotes the index on the feature maps in the horizontal direction, and j denotes the index on feature maps in the vertical direction. Given the value of i of *c−th* channel, coordinate attention firstly iterates elements of the corresponding feature mAP vertically. $y_{c}$ represent the re−weighted feature from *c−th* channel. By utilizing these attention weights, the model can extract the feature of the region of interest in a certain position, which is essential for identification performance.

#### 2.2.3. Network Architecture

It is a fact that the vocalization identification task can be regarded as a speech recognition task since we extracted the 2−D matrix of spectrogram and MFCCs. Here we used the recurrent neural network (RNN) to extract the features from spectrogram and MFCCs. RNN leverages hidden units in which weights come to effect to extract the high dimensional features.

**Figure 5:**
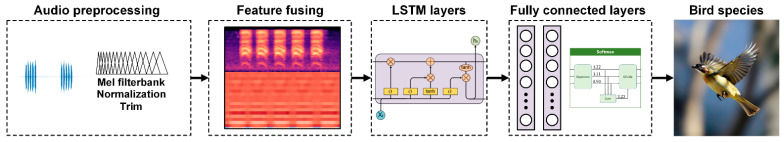
Process of bird species identification.

Bird calls are proved to possess temporal characteristics. Different bird species have their own corresponding rhythmic features. The recurrent neural network was verified to be remarkable in dealing with time−series issues. However, as the time span expanded, the plain RNN failed to learn the long−term context, which is crucial for predicting the status of the next timing. For large training processes, the plain RNN is unable to resolve the problem of gradient disappearance and explosion. Long short−term memory (LSTM) [56] is an advanced variant of RNN which integrates specified gates to retrieve the long or short−term context from the input. Therefore, LSTM was selected as our baseline network (Figure 5). Instead of directly extracting features from spectrogram and MFCCs, LSTM leverages the temporal context to enhance identification performance.

**Figure 6:**
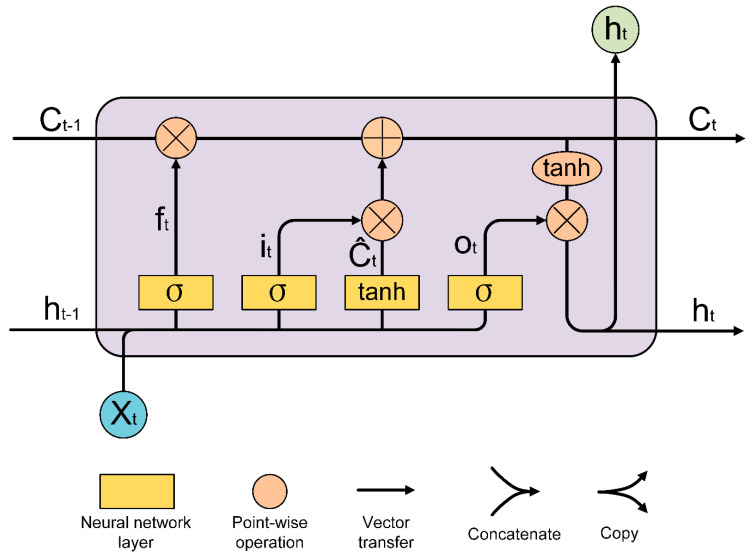
Workflow of LSTM.

A single LSTM module consists of a series of neural networks, point−wise operation, vector transfer, concatenate and copy operation. As shown in Figure 6, the first sigmoid function is regarded as forget gate, which filters the combination of $h_{t-1}$ and $x_{t}$ to retain the useful features as well as discharging deprecated features from the previous cell state $C_{t-1}$. The second sigmoid function and the first tanh function act as input gates collecting the required features that input $x_{t}$ has brought about at time t. Cell state $C_{t}$ represents the cell state at time t, which is the addition of multiplication of the previous cell state and the factorized features from the input $x_{t}$. Depending on the acquired cell state $C_{t}$, the hidden state at time t can be calculated by multiplying the third sigmoid function output of the combination of $h_{t-1}$, $x_{t}$ and the second tanh function output of cell state $C_{t}$. All of the processes of a single LSTM module can be formulated as:

$$f_{t}=\sigma \left(W_{f}\left[h_{t-1},x_{t}\right]+b_{f}\right)$$

$$i_{t}=\sigma \left(W_{i}\left[h_{t-1},x_{t}\right]+b_{i}\right)$$

$${\hat{C}}_{t}=tanh(W_{C}\left[h_{t-1},x_{t}\right]+b_{C})$$

$$C_{t}=f_{t}C_{t-1}+i_{t}{\hat{C}}_{t}$$

$$o_{t}=\sigma \left(W_{o}\left[h_{t-1},x_{t}\right]+b_{o}\right)$$

$$h_{t}=o_{t}tanh(C_{t})$$

where $f_{t}$ regularizes the combination of $h_{t-1}$ and $x_{t}$ for previous cell state, while $C_{t}$ refers to the cell state at time t and integrates features at time *t* as well as features from the previous cell state. $h_{t}$ is the hidden state at time t. $f_{t}$, $i_{t},$ and $o_{t}$ are all neural networks with different parameters inside.

**Figure 7:**
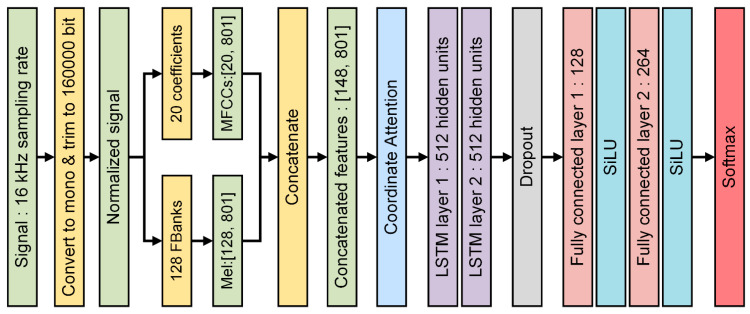
Overview of the proposed deep neural network.

Our proposed LSTM network consisted of six fundamental properties: number of input features, number of output features, number of hidden units, number of layers, dropout rate, and activation function. In order to reduce the computation cost and accelerate the training process, the number of input features was configured at 148. Due to the number of bird species considered in this paper, we set the number of output features to 264. For better learning performance, each layer’s hidden units were set to 512. In order to avoid overfitting during the training process, the dropout function was activated, and the dropout rate was set to 0.3 for sure. Instead of directly implementing rectified linear unit (ReLU) function, the advanced activation function sigmoid linear unit (SiLU) [57] was in our consideration to avoid the inactivation of neurons inside the LSTM and fully connected layers. As LSTM layers produced the output, we fed it into two fully connected layers with the SiLU activation function. The output dimension of fully connected layers was 264 with respect to the number of species. Finally, we used the Softmax [30] function to output the final prediction of bird species (Figure 7).

## 3. Results

### 3.1. Evaluation Metrics

In order to evaluate our model’s performance, a set of metrics was introduced. Accuracy, precision, recall, and F1 score are defined as:

$$Accuracy=\frac{correct\;classifications}{all\;classifications}$$

$$Precision=\frac{TP}{TP+FP}$$

$$Recall=\frac{TP}{TP+FN}$$

$$F_{1}=\frac{2\cdot Precision\cdot Recall}{Precision+Recall}$$

where TP denotes the number of true positives, TN denotes the number of true negatives, FP denotes the number of false positives, and FN denotes the number of false negatives.

Accuracy is used to measure the conventional performance of a single bird species considering correct predictions and all predictions. Precision merely measures the performance of avoiding false predictions but ignoring the missing predictions of true positives, while recall is concerned with the opposite of precision. The F1−score is commonly used to assess the model’s dichotomous performance, which considers both precision and recall. AUC is selected as an additional metric to evaluate the performance, which is defined as:

$$AUC=\frac{\sum_{t_{0}\in D^{0}}\sum_{t_{1}\in D^{1}}I[f\left(t_{0}\right)<f\left(t_{1}\right)]}{\left|D^{0}\right|\cdot \left|D^{1}\right|}$$

where $I[f\left(t_{0}\right)<f\left(t_{1}\right)]$ denotes an indicator function that returns 1 if $f\left(t_{0}\right)<f\left(t_{1}\right)$ otherwise, return 0; $D^{0}$ is the set of negative samples, and $D^{1}$ is the set of positive samples. The top−5 accuracy was adopted since our dataset contains a large number of classes. It is a smoother metric than the conventional top−1 accuracy. The top−5 accuracy is defined as:

$$\mathrm{Top}\text{-}5\;\mathrm{Accuracy}\left(y,\hat{f}\right)=\frac{\sum_{i=0}^{n_{\mathrm{samples}}-1}\sum_{j=1}^{5}I\left({\hat{f}}_{i,j}=y_{i}\right)}{n_{\mathrm{samples}}}$$

where i is the index of n size samples, j represents the j−th largest prediction score, $y_{i}$ represents the corresponding ground truth of i−th sample, ${\hat{f}}_{i,j}$ is the prediction of i−th sample at j−th largest prediction score, and $I\left({\hat{f}}_{i,j}=y_{i}\right)$ is an indicator function.

Here we used the micro mAP metric to evaluate the overall performance of our model for all bird species, which is defined as:

$$mAP=\frac{\sum_{k=1}^{N}AvgP\left(k\right)}{N}$$

where k represents the index of bird species, N represents the total quantity of bird species, AvgP(k) represents the average precision of k−th bird species, which is defined as:

$$AvgP\left(k\right)=\overset{S_{k}}{\underset{s=1}{\sum }}P\left(k_{s}\right)\Delta R\left(k_{s}\right)$$

where $S_{k}$ is the total number of samples of k−th bird species, $P\left(k_{s}\right)$ represents the P(k) at the first s samples of k−th bird species, $\Delta R\left(k_{s}\right)$ represents the amount change in recall between the first s−1 and the first s samples of k−th bird species.

### 3.2. Experimental Setup

In this paper, the training and testing process was carried out in the following environment given in Table 1.

In terms of the data, we divided them into a training set and a test set in a 4:1 ratio. Each class kept the same train–test ratio as we utilized the stratified sampling. Before activating the training process, several essential hyper−parameters were configured properly, which are manifested in Table 2.

Since our method considered multi−species identification, the cross−entropy function was selected to compute the loss, which can be formulated as:

$$loss_{CE}=-\overset{N}{\underset{n=1}{\sum }}y_{n}log{\hat{y}}_{n}+\left(1-y_{n}\right)log(1-{\hat{y}}_{n})$$

where n denotes the index of bird species, N represents the total number of bird species. $y_{n}$ denotes the ground truth of bird species and ${\hat{y}}_{n}$ denotes the prediction of bird species by the model.

### 3.3. Performance of the Proposed Method

#### 3.3.1. Effectiveness of Feature Type

**Figure 8:**
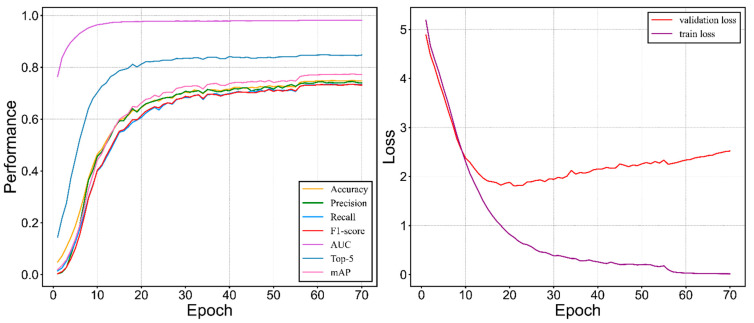
Performance of the proposed model.

The proposed model was trained for 70 epochs (Figure 8). According to the conclusion of the prior research on identification based on acoustic features [58], the input acoustic feature can bring crucial effects on the accuracy of the model. Therefore, we firstly conducted a feature validation experiment in which multiple types of input features were assigned to our proposed method to generate the corresponding results listed in Table 3. Apart from that, normalization is a function that compares the features of different dimensions numerically, which is conducive to improving the model’s accuracy. As a result, the search process of the optimal gradient becomes smoother, which is hopeful for model training to converge to an ideal solution. For the feature type of Mel−spectrogram and MFCCs, we assigned normalization to both of them to measure the potential improvement that normalization exerted. The normalization function is formulated as:

$$NormFeats=\frac{input-mean\left(input\right)}{std\left(input\right)}$$

where mean() function is used to transform all elements of input feature tensor into mean value while std() function generates the standard deviation of input feature tensor.

**Figure 9:**
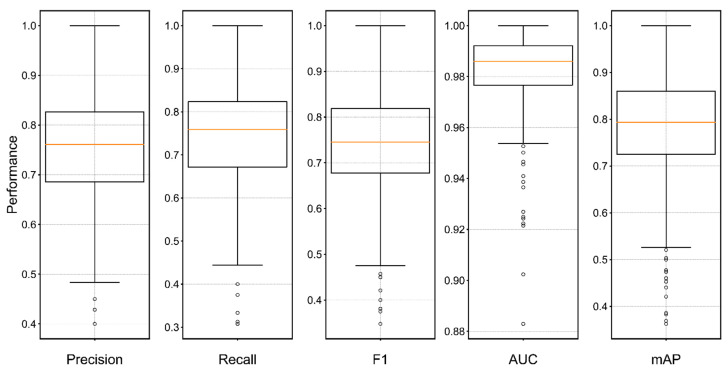
Boxplots of the proposed model. Each metric includes 264 results. Statistics: *Pre_max_* = 100.00%, *Pre_min_* = 40.02%, *Pre_std_* = 0.1170, *Pre_avg_* = 74.51%; *Rec_max_* = 100.00%, *Rec_min_* = 30.76%, *Rec_std_* = 0.1268, *Rec_avg_* = 73.51%; *F1_max_* =100.00%, *F1_min_* = 34.78%, *F1_std_* = 0.1090, *F1_avg_* = 73.45%; *AUC_max_* = 100.00%, *AUC_min_* = 88.29%, *AUC_std_* = 0.0163, *AUC_avg_* = 98.21%; *mAP_max_* = 100.00%, *mAP_min_* = 36.25%, *mAP_std_* = 0.1195, *mAP_avg_* = 77.43%.

**Figure 10:**
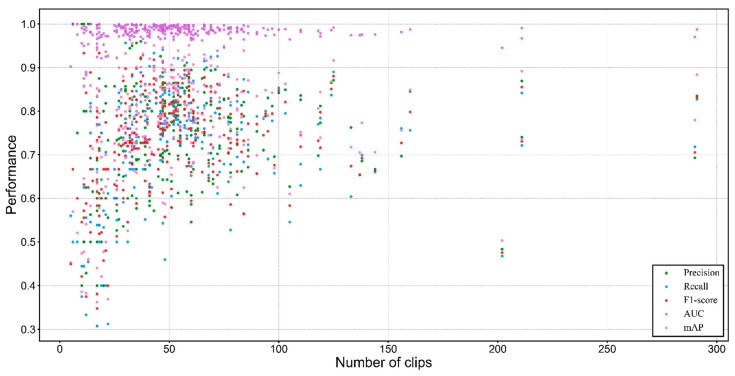
Distribution of performance scores by data volume.

**Figure 11:**
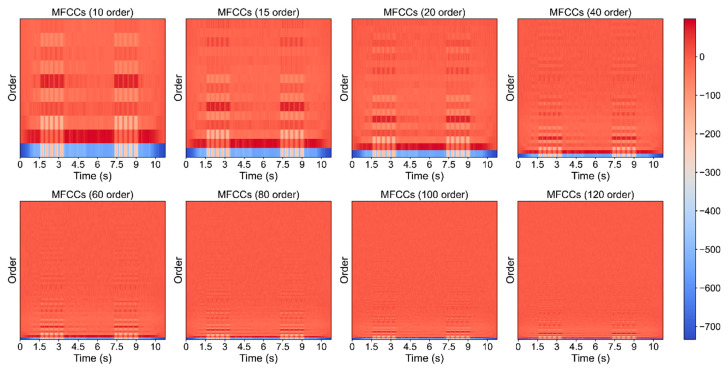
Comparison of multiple orders of MFCCs.

**Figure 12:**
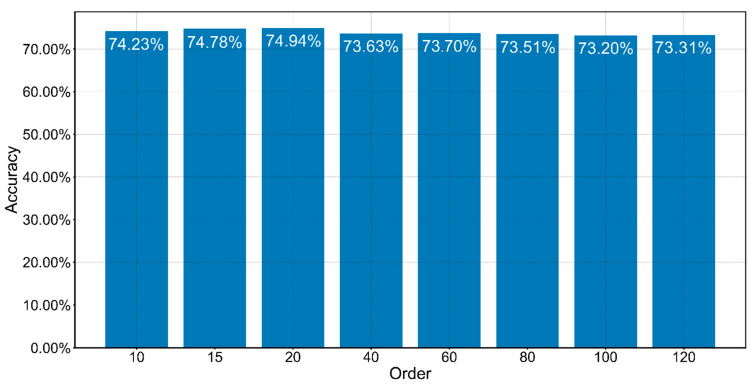
Order impact of MFCCs on the performance of the proposed model.

Our proposed model achieved a good convergence at around 68 epoch (Figure 8). It maintained acceptable standard deviations of five metrics (precision, recall, F1−score, AUC, and mAP). The number of flyer dots is relatively small and is centered in a low-performance area which implicitly indicates that our model maintained an improved identification performance for the vast majority of bird species (Figure 9). There is a trend that performance is generally positively correlated with the amount of bird calls datasets (Figure 10). The waveform is a kind of raw graph that only presents the amplitude changes in time order regardless of the distribution of frequencies. It was evident that waveform possessed the lowest performance in all metrics. Mel−spectrogram generally outperformed a waveform, especially in mAP but below MFCCs. More importantly, MFCCs achieved the best performance in all metrics compared to other non−combined feature types. Compared with the original combined feature from Mel−spectrogram and MFCCs, the model generated by normalized Mel−spectrogram and MFCCs was significantly improved by about 4.00% accuracy from 70.94% to 74.94%. From the results in Table 3, it is apparent that both Mel−spectrogram and MFCCs play crucial roles in leveraging the model’s performance. As the feature structure of MFCCs is determined by its orders, an experiment on different orders of MFCCs was conducted to find the best set of orders (Figure 11). Our proposed method generated the best model using the 20 orders of MFCCs feature, which achieved about 74.94% accuracy (Figure 12).

#### 3.3.2. Model Comparison

From the results elaborated above, the combined Mel−spectrogram and MFCCs were highlighted among all other types of input features. In order to explore the effectiveness of the proposed network structure, a comparison experiment was carried out to measure the performance when using multiple methods, including CNN, support vector machine (SVM) [59], random forest (RF) [60], Fisher’s linear discriminant analysis (FLDA) [61], k−nearest neighbors (k−NN) [62], and gated recurrent unit (GRU, Chung) [63]. All of the methods were conducted using fused Mel−spectrogram and MFCCs features to assess the robustness of these methods, respectively. For the sake of computational restriction when running traditional machine learning techniques, we reduced the dimension of the input audio clips from 148 × 801 to 1 × 15,087. The results in Table 4 demonstrate that the proposed method achieved the best performance in six out of seven metrics.

#### 3.3.3. Effectiveness of Inner Modules

To measure the effects of different modules of the proposed method, we conducted an ablation study on the inner modules of the proposed model. The number of layers was doubled for grasping more fine−grained temporal features. We generated three models by training LSTM, LSTM with SiLU, and LSTM with coordinate attention. For a standalone LSTM model, we tested multiple numbers of hidden units, and the LSTM of 512 hidden units gained a higher score. The results are shown in Table 5. The accuracy increased by about 0.46% from 72.29% to 72.75% when utilizing SiLU and about 2.65% from 72.29% to 74.94% when utilizing both CA and SiLU.

#### 3.3.4. Visualization of Features

**Figure 13:**
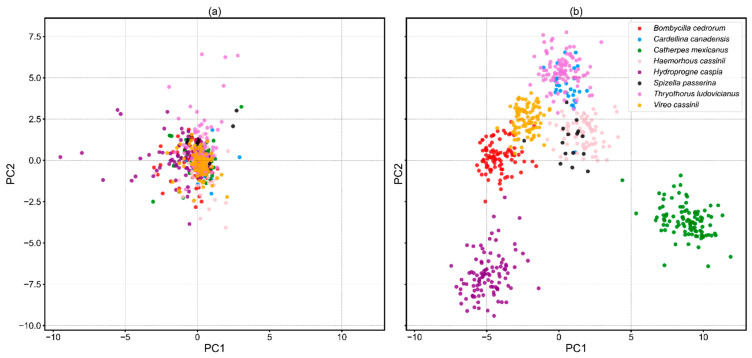
PCA on features of multiple types. (**a**) PCA on raw features (**b**) PCA on learned features.

To describe the visual traits of the input feature and output feature, principal component analysis (PCA) [64] was implemented to reduce the dimension of these two feature types, respectively. The parameter component was set to 2, which is essential for 2−D visualization. The input feature originated from both Mel−spectrogram and MFCCs in a matrix of 148 × 801, while the output feature was generated in the vector of 264 from the process stack of coordinate attention, LSTM layers, and fully connected layers. We randomly selected 600 samples out of eight bird species. From the view of the PCA result (Figure 13), the samples of input features were scattered densely. In contrast, the samples of output features were generally divided into two clusters, which implicitly illustrated the good classification performance of the model.

#### 3.3.5. ROC Illustration

**Figure 14:**
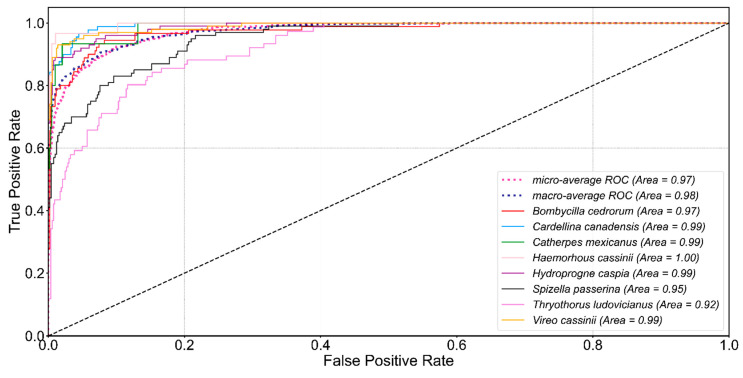
ROC Curves of the selected eight bird species. Micro and macro average ROC curves are dotted lines.

In order to present a diagnostic performance of the proposed method, the receiver operating characteristic (ROC) [65] curve was introduced. Six hundred test samples out of eight species were tested for rendering a single−class binary classification ROC curve (Figure 14). The curve was generated by plotting the true positive rate (TPR) against the false positive rate (FPR) at various threshold settings. The proposed model maintained a good diagnostic ability and achieved 0.97, and 0.98 micro and macro averaged AUC.

## 4. Discussion

### 4.1. Revelation of the Proposed Model

The performance of the deep learning model is conventionally dominated by its quantity of training datasets [66]. We illustrated a distribution of performance scores of different metrics considering the amount of validation bird calls dataset (Figure 10). Overfitting is the main issue that greatly limits the overall performance of the model. Therefore, we enlarged our training dataset as much as possible to mitigate that issue. Our dataset contains 72,172 bird call clips (126 gigabytes), which is far more than prior studies [35,37]. A large dataset ensures the baseline performance of the model. Moreover, the dropout rate was empirically experimented with many times to find the best set of it that ultimately minimizes the overfitting problem. We explored the importance of multiple types of input features to validate MFCCs and Mel−spectrogram. The experiment results presented that MFCCs played an important role in boosting the overall performance of the model, and thus, we discussed the effect of MFCCs orders on the model.

The CA [55] module was implemented on the input feature that was fused before because the merged input feature (Mel−spectrogram and MFCCs) was already full of semantic elements. It was needless to generate additional convolution kernels or pooling layers to fine−tune it as we want the LSTM module to extract more fine−grained temporal features. Moreover, the CA module is able to parallelly capture both the channel−wise and positional context, which is ignored in the convolutional block attention module (CBAM) [67]. In terms of LSTM structure, considering the 264 involved bird species, we empirically set the number of hidden units to 512 to process the input feature (148 × 801). We noticed that the 512 is probably the best set of the hidden units, which is better than the set of 256 and the set of 1024. We also found an issue out of expectation that the bidirectional LSTM [68] performed worse, which might be caused by overfitting. The activation function is the critical component in the model training process. We chose SiLU as the activation function, which is able to smooth the gradient of neurons whose value is below zero. The derivative of layers close to the output layer becomes very small and reaches zero, resulting in the weight of the layers near the output layer updating slowly or even not updating, and thus these layers learn about nothing [69]. In this way, our model is able to mitigate the invalidation of neurons during the training process. We experimented with validating the CA and SiLU. It showed that the model achieved the highest performance by leveraging both CA and SiLU. Empirically, the deep learning model is sensitive to hyper−parameters. In order to avoid overfitting, the learning rate was set to as low as possible. We set the hyper−parameter ‘epoch’ to 70 to achieve ideal performance.

We conducted a comparison among multiple traditional machine learning models and other deep learning models (CNN, GRU, and our proposed model). These models presented different performances, but they can be divided into two groups: The proposed model, GRU, CNN, and Random Forest, which displayed good performance in all metrics compared to the rest of the models; SVM, k−NN, and FLDA, which showed unsatisfactory performance.

### 4.2. Advantages of Our Work

The ablation experiment demonstrated that our model excelled over many other methods, such as SVM and GRU. Our proposed model significantly outperformed other mainstream machine learning models, and the model’s performance scores in all metrics are stable compared to RF, which gained the highest precision, but the scores of other metrics are relatively low. We novelly proposed the fused features from Mel−spectrogram and MFCCs as input for the model, which presented a good improvement of performance. We proposed a novel preprocessing method for identifying bird species based on their calls. The preprocessed bird call clips are more robust than the original raw dataset. Notably, our work considered seven metrics to evaluate models’ performance and novelly introduced the top−5 accuracy metric in our study. The top−5 accuracy is considerable when the classes of bird species are relatively large, and it is a smoother metric than top-1 accuracy, which helps to evaluate models more comprehensively. Our proposed model displayed a competitive performance of 84.65% top−5 accuracy. When researchers are coping with challenging recordings of a certain species which tend to be misidentified by the machine learning model, they could reference the top−5 accuracy result to make a final prediction incorporating the expertise or meta−data of the target bird species. The performance of our model in mAP is very close to the state−of−the−art BirdNET [43], which achieved 79.1% mAP regarding 984 bird species.

### 4.3. Limitations and Future Improvements

We found that AUC is an indistinctive metric compared to other metrics when the number of bird species is massive. Our model might be sensitive to the volume of the dataset, so before realizing a model regarding a real scene application of bird diversity survey, it will take some time to retrieve data and conduct data cleaning. Although our model is very close to the state−of−the−art model BirdNET, there is potential space to improve the model. We plan to further improve our model by leveraging the knowledge distillation and conducting more experiments on multiple features and model parameters.

## 5. Conclusions

In this paper, we collected a huge amount of bird calls out of 264 bird species from Xeno−Canto and proposed an efficient deep learning method to identify these bird species based on their acoustic features. We proposed a pre−processing method for audio clips and a method for input feature fusing. The proposed method achieved 77.43% mAP and maintained a good generalization ability. The proposed model was evaluated, and our work might be useful for bird species identification and avian biodiversity monitoring.

## Figures and Tables

**Table 1:** Training and testing environment.

Environment	Setup
GPU	NVIDIA GeForce RTX 3080 Ti 12 G
CPU	Intel(R) Core(R) i9−12900 KF 3.90 GHz
RAM	64 G
Operating system	Windows 11 64 bit
Software environment	Python 3.8.10, Pytorch 1.12, CUDA 11.6

**Table 2:** Training settings.

Hyper−Parameter	Value
Epoch	70
Batch size	256
Learning rate	0.0001
Learning rate decay	0.1 per 10 epochs
Optimizer	AdamW
Loss function	Cross entropy

**Table 3:** Performance of our proposed method when different features are given as input. The best results are shown in bold font.

Feature Type	Accuracy	Precision	Recall	F1−Score	AUC	Top−5	mAP
Waveform	43.59%	41.76%	35.80%	35.93%	95.22%	69.02%	40.29%
Mel−spectrogram	68.60%	68.98%	67.23%	67.12%	97.50%	82.19%	71.23%
MFCCs (20 order)	68.84%	66.63%	66.92%	66.63%	97.58%	82.47%	70.81%
Mel−spectrogram and MFCCs	70.94%	70.98%	69.41%	69.36%	97.91%	83.64%	72.72%
Normalized Mel−spectrogram	72.39%	72.69%	70.68%	70.86%	97.96%	84.04%	74.68%
Normalized MFCCs	72.61%	73.35%	71.25%	71.57%	98.01%	84.14%	75.01%
Normalized Mel and MFCCs	**74.94%**	**74.51%**	**73.51%**	**73.45%**	**98.21%**	**84.65%**	**77.43%**

**Table 4:** Performance comparison between the proposed method, CNN, SVM, RF, FLDA, KNN, and GRU. The best results are shown in bold font.

Method	Accuracy	Precision	Recall	F1−Score	AUC	Top−5	mAP
CNN (ResNet−101)	66.47%	66.79%	64.32%	64.42%	97.53%	81.84%	68.67%
SVM	22.16%	42.01%	16.39%	19.51%	89.15%	49.94%	25.79%
RF	52.84%	81.58%	53.14%	62.02%	92.28%	65.57%	58.54%
FLDA	17.01%	17.39%	17.69%	16.58%	78.43%	38.24%	11.52%
k−NN	11.12%	10.66%	10.64%	9.75%	75.33%	48.32%	11.07%
GRU	71.72%	71.40%	69.77%	70.01%	97.66%	83.07%	72.52%
The proposed method (Ours)	**74.94%**	74.51%	**73.51%**	**73.45%**	**98.21%**	**84.65%**	**77.43%**

**Table 5:** Ablation study of the proposed method. The coordinate attention and SiLU module were disassembled in the ablation experiment. The best results are shown in bold font.

Module	Accuracy	Precision	Recall	F1−Score	AUC	Top−5	mAP
LSTM (256 hidden units)	67.19%	67.57%	64.99%	65.19%	97.63%	82.35%	69.60%
LSTM (512 hidden units)	72.29%	70.89%	70.85%	70.18%	97.94%	83.48%	74.52%
LSTM (1024 hidden units)	70.81%	71.11%	69.18%	69.03%	97.89%	83.75%	73.29%
Bi−LSTM (512 hidden units)	68.51%	67.66%	67.25%	66.38%	97.55%	82.38%	70.82%
LSTM with SiLU	72.75%	72.00%	71.19%	70.82%	97.94%	84.04%	74.80%
LSTM with CA and SiLU	**74.94%**	**74.51%**	**73.51%**	**73.45%**	**98.21%**	**84.65%**	**77.43%**

## Data Availability

Tabulated data of bird calls are available in the Supplementary Materials. Raw data are available upon request from the corresponding author.

## Supplementary Materials

The following supporting information can be downloaded at: https://www.mdpi.com/article/10.3390/ani12182434/s1, Supplementary Material S1: Tabulated data of bird calls.
